# Three-dimensional imaging mass cytometry for highly multiplexed molecular and cellular mapping of tissues and the tumor microenvironment

**DOI:** 10.1038/s43018-021-00301-w

**Published:** 2021-12-24

**Authors:** Laura Kuett, Raúl Catena, Alaz Özcan, Alex Plüss, Cancer Grand Challenges IMAXT Consortium, H. R. Ali, M. Al Sa’d, S. Alon, S. Aparicio, G. Battistoni, S. Balasubramanian, R. Becker, B. Bodenmiller, E. S. Boyden, D. Bressan, A. Bruna, Marcel Burger, C. Caldas, M. Callari, I. G. Cannell, H. Casbolt, N. Chornay, Y. Cui, A. Dariush, K. Dinh, A. Emenari, Y. Eyal-Lubling, J. Fan, A. Fatemi, E. Fisher, E. A. González-Solares, C. González-Fernández, D. Goodwin, W. Greenwood, F. Grimaldi, G. J. Hannon, S. Harris, C. Jauset, J. A. Joyce, E. D. Karagiannis, T. Kovačević, L. Kuett, R. Kunes, A. Küpcü Yoldaş, D. Lai, E. Laks, H. Lee, M. Lee, G. Lerda, Y. Li, A. McPherson, N. Millar, C. M. Mulvey, I. Nugent, C. H. O’Flanagan, M. Paez-Ribes, I. Pearsall, F. Qosaj, A. J. Roth, O. M. Rueda, T. Ruiz, K. Sawicka, L. A. Sepúlveda, S. P. Shah, A. Shea, A. Sinha, A. Smith, S. Tavaré, S. Tietscher, I. Vázquez-García, S. L. Vogl, N. A. Walton, A. T. Wassie, S. S. Watson, J. Weselak, S. A. Wild, E. Williams, J. Windhager, C. Xia, P. Zheng, X. Zhuang, Peter Schraml, Holger Moch, Natalie de Souza, Bernd Bodenmiller

**Affiliations:** 6 Cancer Research UK Cambridge Institute, Li Ka Shing Centre, University of Cambridge, Cambridge, UK; 7 Institute of Astronomy, University of Cambridge, Cambridge, UK; 8 McGovern Institute, Departments of Biological Engineering and Brain and Cognitive Sciences, Massachusetts Institute of Technology, Cambridge, MA, USA; 9 Department of Molecular Oncology, BC Cancer, part of the Provincial Health Services Authority, Vancouver, British Columbia, Canada; 10 Department of Pathology and Laboratory Medicine, University of British Columbia, Vancouver, British Columbia, Canada; 11 Department of Chemistry, University of Cambridge, Cambridge, UK; 12 Súil Interactive Ltd, Dublin, UK; 13 Department of Oncology and Cancer Research UK Cambridge Institute, University of Cambridge, Cambridge, UK; 14 Herbert and Florence Irving Institute for Cancer Dynamics, Columbia University, New York, NY, USA; 15 Howard Hughes Medical Institute, Department of Physics and Department of Chemistry and Chemical Biology, Harvard University, Cambridge, MA, USA; 16 Department of Oncology and Ludwig Institute for Cancer Research, University of Lausanne, Lausanne, Switzerland; 17 Computational Oncology, Department of Epidemiology and Biostatistics, Memorial Sloan Kettering Cancer Center, New York, NY, USA; 18 Department of Computer Science, University of British Columbia, Vancouver, BC, Canada; 19 New York Genome Center, New York, NY, USA; 2 Institute of Molecular Health Sciences, ETH Zurich, Zürich, Switzerland; 3 Leica Geosystems part of Hexagon, Heerbrugg, St. Gallen, Switzerland; 4 Department of Pathology and Molecular Pathology, University Hospital Zurich, Zurich, Switzerland; 5 Institute of Molecular Systems Biology, ETH Zurich, Zürich, Switzerland

## Abstract

A holistic understanding of tissue and organ structure and function requires the detection of molecular constituents in their original three-dimensional (3D) context. Imaging mass cytometry (IMC) enables simultaneous detection of up to 40 antigens and transcripts using metal-tagged antibodies but has so far been restricted to two-dimensional imaging. Here we report the development of 3D IMC for multiplexed 3D tissue analysis at single-cell resolution and demonstrate the utility of the technology by analysis of human breast cancer samples. The resulting 3D models reveal cellular and microenvironmental heterogeneity and cell-level tissue organization not detectable in two dimensions. 3D IMC will prove powerful in the study of phenomena occurring in 3D space such as tumor cell invasion and is expected to provide invaluable insights into cellular microenvironments and tissue architecture.

Tissues and organs are complex ecosystems consisting of numerous cell types arranged in a manner that is inextricably related to function. Understanding tissue function and pathology thus requires knowledge of constituent cells and their states, extracellular matrix proteins and vasculature in the context of their native 3D arrangement. Historically, tissues have been studied using microscopy and recently developed methods have enabled various types of 3D tissue analysis (Supplementary Table 1). Confocal 3D microscopy enables analysis of tissue sections at subcellular resolution but is limited to a depth of about 100 μm^1^. Multi-photon confocal and light-sheet microscopes allow for 3D reconstructions of up to 1-mm tissue depth at single-cell resolution^2,3^. As these 3D microscopy methods rely on fluorescent reporters that show high spectral overlap, the number of epitopes that can be measured simultaneously is limited.

To enable multiplexed tissue analysis, cyclic immunostaining and chromogenic approaches have been used^4–7^ and such methods have also been implemented in 3D^8,9^. In addition to fluorescence-based approaches, mass spectrometry-based imaging of epitopes and transcripts is becoming broadly used. In mass spectrometry-based technologies, mass tags, such as a molecule of a defined mass or metal isotopes, are used as reporters on affinity reagents^10,11,12^. IMC allows simultaneous detection of up to 40 antigens^13^ and nucleic acid sequences^14^ in formalin-fixed paraffin-embedded (FFPE) tissues^15^, in frozen tissue sections^16^ and in cultured cells^17^. Currently, however, none of these methods combines multiplex detection of many targets with 3D tissue imaging, which is necessary for visualization of single cells together with larger structures such as blood vessels.

Here we describe an extension of IMC to the analysis of tissues in 3D. With the 3D IMC method, the volume and depth of a tissue that can be analyzed is limited mainly by the measurement time. The full pipeline, from sample processing to cell-level computational analysis of a complete 3D model, can be performed in 1 week^15,18^. We demonstrate how 3D IMC enables the study of tumor architecture by combining the analysis of tissue volumes with single-cell information. We show that spatial heterogeneity of marker expression and preferential cell–cell interactions become apparent with 3D models and that spatially contained events could be captured within a single 3D model. Overall, we demonstrate that the detailed models generated with 3D IMC facilitate comprehensive, single-cell-resolution analysis of cellular microenvironments and tissue architecture.

## Results

### Generation of 3D models from IMC data

**Extended Data Fig. 1:**
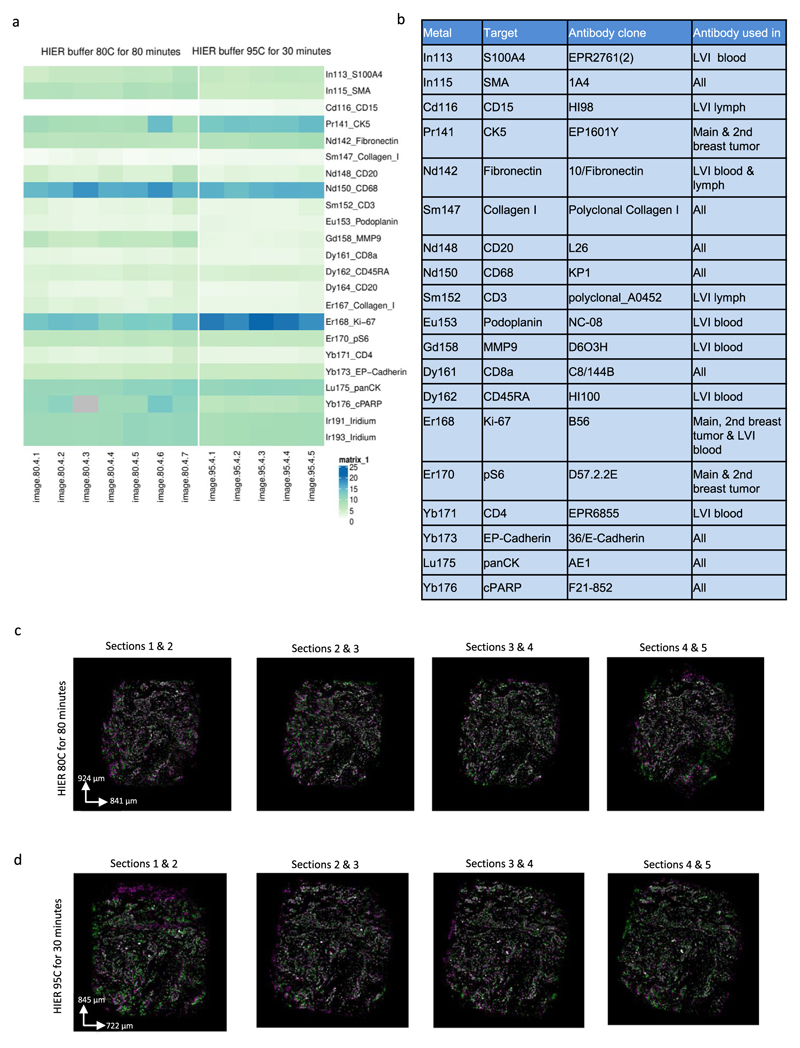
A comparison of 95°C and 80°C heat-induced antigen retrieval for consecutive 2-μm slices from a breast cancer sample (Methods). (a) Signal-to-noise ratio was calculated based on the ratio of metal ion counts within a rough 2D nuclear segmentation mask to background signal for 2um-thick consecutive slices from the same breast cancer sample (n = 1) that either underwent antigen retrieval at 80 °C for 80 minutes (7 slices) or at 95°C for 30 minutes (5 slices) as part of a one experiment. The first five consecutive images are shown in c) and d). The experiment also included a second independent sample (data not shown). (b) Antibody clones used for antigen retrieval comparison. (c) and (d) Overlays of the pairwise consecutive slices aligned with affine transformation after antigen retrieval at c) 80 °C (images shown for the first 5 slices) and d) 95 °C (images shown for all the slices). Green and magenta indicate two consecutive slices, and white shows overlapping region. Overlapping images were visualized in ImageJ2.

**Fig. 1:**
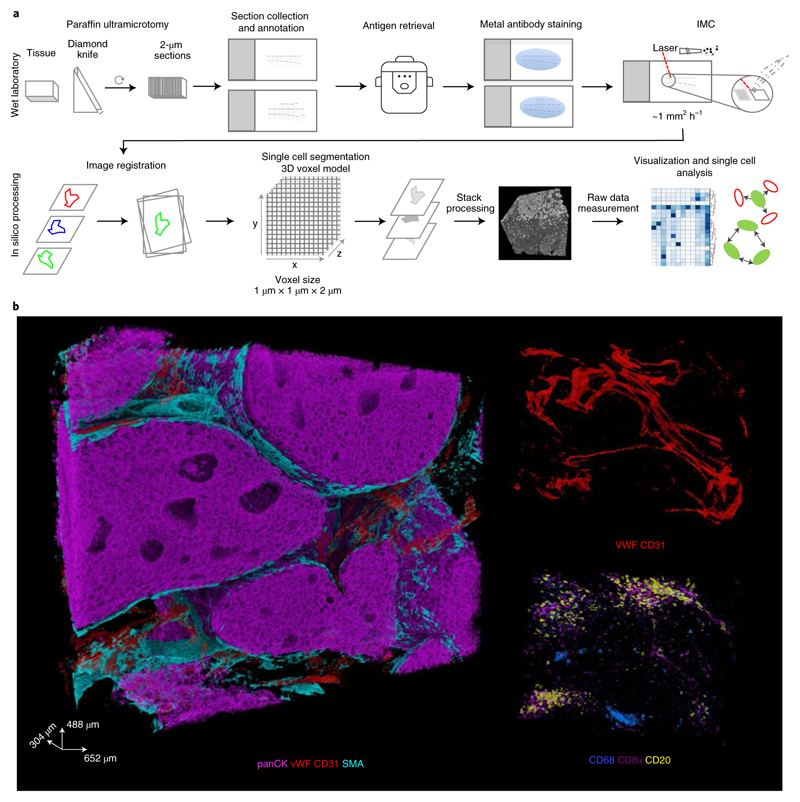
Experimental and computational workflow for 3D IMC. **a**, Small rods or blocks of FFPE tissue are cut into 2-**μ**m sections with an ultramicrotome and a modified diamond knife. Sequential sections were collected on regular microscopy slides. Typically, 20 to 40 sections were placed on each glass slide. After rehydration, tissues were subjected to antigen retrieval, followed by staining with metal-labeled antibodies. All sections were analyzed by IMC. Data were processed computationally to order sections according to the annotation. Images are aligned and cells are segmented with a 3D watershed algorithm. Finally, a full 3D model can be analyzed both at the voxel and cell level. **b**, Examples of raw data voxel rendering for the indicated markers in a representative example from one out of the two breast carcinoma 3D IMC models. AGAVE 1.0.0.1 was used for 3D rendering of the data.

Our 3D IMC approach relies on serial sectioning of a tissue cylinder punched from a paraffin-embedded tissue. We optimized our sample processing methods and settled on using an ultramicrotome with a diamond knife designed for FFPE sectioning to minimize deformations that are known to be caused during tissue cutting, handling of thin slices, and further experimental procedures^19^. We chose to cut 2-μm-thick sections to provide a compromise between capturing single cells across multiple slices and the difficulty of handling ultra-thin slices, thereby making the approach accessible to a broader user base. Tissue sectioning is followed by tissue hydration and heat-induced epitope retrieval (Extended Data Fig. 1). After acquiring two-dimensional (2D) IMC data, we assemble 3D tissue models and derive single-cell marker profiles using a computational workflow that we established based on publicly available tools (Fig. 1a). Data acquisition for 0.5 mm^3^ currently takes about 1 week.

**Extended Data Fig. 2:**
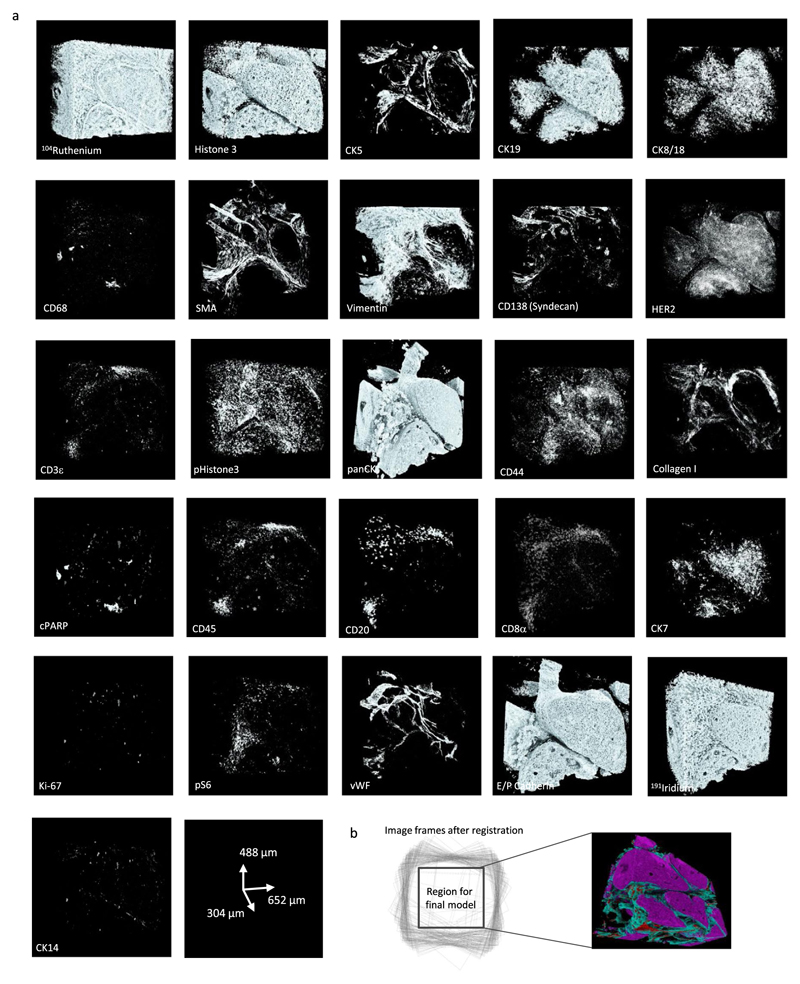
Single-channel marker expression in a breast carcinoma 3D IMC model. Raw data voxel rendering of different channels for a breast carcinoma 3D IMC model. AGAVE 1.0.0.1 was used for 3D rendering of the data. (b) The final model size is chosen to ensure that the 3D volume is generated from an area that overlaps between all the slices in the stack.

The 3D IMC approach enables the study of the spatial distribution of around 40 epitopes in the context of 3D tissue architecture. To exemplify the 3D IMC workflow, we reconstructed 3D models from different breast cancer samples. For the main model with a final size of 652 × 488 × 304 μm^3^, we used an HER2^+^ ductal breast carcinoma sample (Fig. 1b). We cut 152 consecutive slices from the tissue sample and stained the sections with a breast-cancer-centric panel of antibodies (Supplementary Table 2 and Extended Data Fig. 2a) designed to reveal basal and luminal cells, vascularization, immune cell infiltration, proliferation, apoptosis, hypoxia, cell signaling and collagen deposition. After data acquisition, we converted the files to TIFF format and aligned consecutive images to achieve the final model from the overlapping area of the images (Extended Data Fig. 2b and Supplementary Videos 1 and 2). The raw voxel-level data rendering of this sample shows regions positive for pan-cytokeratin (panCK) intertwined with blood vessels and stromal mass (Fig. 1b and Supplementary Video 3). The stromal compartment seems heterogeneous in cell composition throughout the volume analyzed, expressing common immune cell markers, including CD20, CD8a, CD3 and CD68 (Fig. 1b). Thus, with 3D IMC, spatial arrangement of multiple markers can be analyzed throughout the tissue volume by evaluating distributions of the raw voxel-level data.

### Derivation of phenotypic single-cell data from 3D IMC images

**Extended Data Fig. 3:**
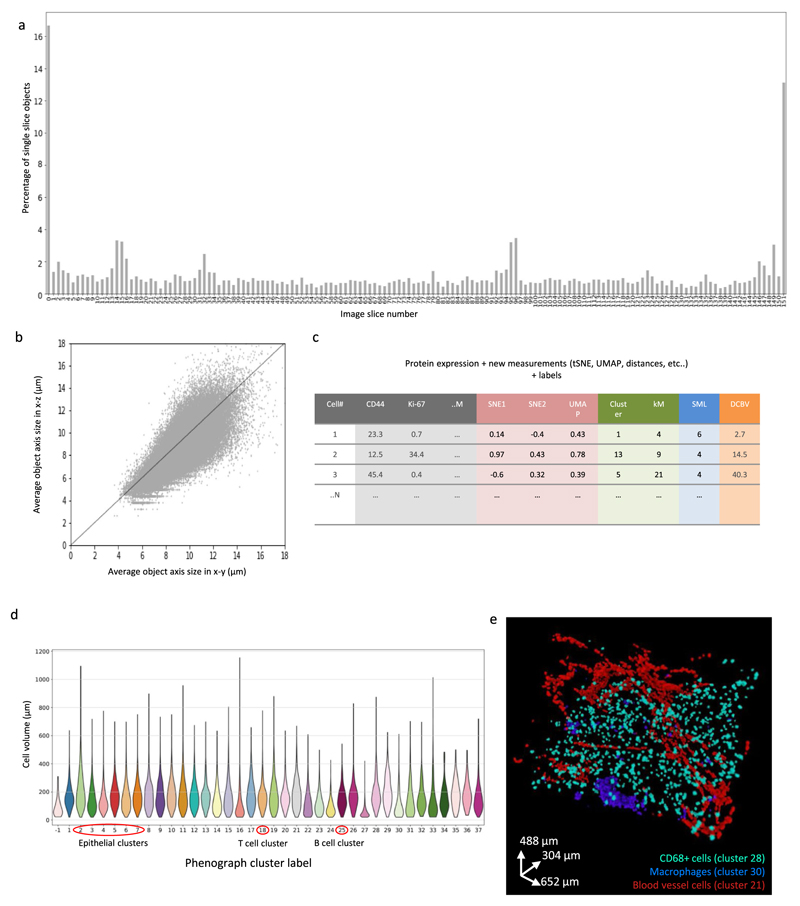
Evaluation of the 3D single cell segmentation for a breast carcinoma 3D IMC model. All panels show data from the same breast carcinoma sample shown in Figs. 1 and 2. (a) Percentage of objects occurring only on one slice for the full 152-slice stack after 3D segmentation to evaluate if the final cell masks were affected by the intensity variation between the consecutive slices. (b) Average axis length for each segmented object in x-y plane and in y-z plane. The z axis was calculated with 2-μm resolution. Axis length was calculated as the averages of minor and major axes using skimage.mesure.regionprops function^37^. (c) Schematic showing the application of multiple methods to augment and annotate the catalog (*N* cells, *M* channels). The data table includes cluster identifiers and dimensionality reduction coordinates. Topographic information such as distance to a particular structure can be calculated by combining the 3D mask with other calculated masks, for instance, the distance to closest blood vessel. (d) Cell volumes calculated as total number of voxels for each segmented object for each cell cluster identified by Phenograph. (e) Distribution of CD68 + clusters with cluster 28 in green and cluster 30 in blue, and cluster 21 in red (endothelial cells). AGAVE 1.0.0.1 was used for 3D rendering of the data.

**Fig. 2:**
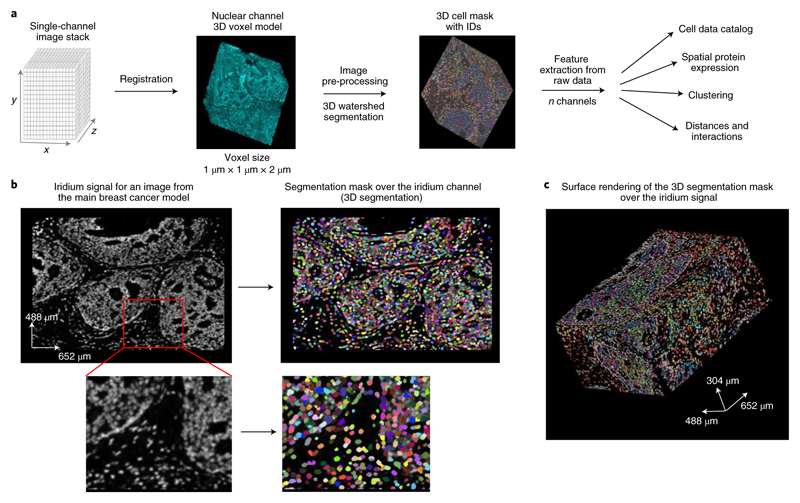
Single-cell segmentation of a 3D IMC generated breast carcinoma model. **a,** Segmentation and cell catalog generation pipeline. A voxel model is used as an input for a 3D watershed segmentation. Detected objects are assigned unique cell identifiers. Statistics for all channels and morphological descriptors are calculated for every segmented cell (*N* cells, *M* channels). **b**, Iridium signal from a single representative slice (no. 58 out of 152) from one out of the two breast carcinoma models used in this study, the same model is also displayed in Fig.1b (left). Segmentation mask overlay of the iridium channel (right). In the segmentation mask, different cells are labeled with random colors. **c**, Example of a full segmentation mask over the surface-rendered iridium channel in the same model (containing a total of 152 slices). Images were produced with napari v.0.4.1rc2 (ref. ^40^).

To study the tumor ecosystem in more depth, we extracted single-cell information from the overlapping area of the images using a 3D watershed segmentation (Fig. 2a,b). Segmentation assigns each cell a unique identifier enabling the computation of antibody signal statistics and spatial data (such as volume and direct neighbor interactions) for each single cell. The antibody signal statistics, hereafter referred to as marker expression, is calculated as the mean intensity of ion counts over each single-cell 3D mask for every channel in the antibody panel. We performed the 3D watershed segmentation on the nuclear channel stained with iridium after applying a set of digital image processing steps (Methods). The segmentation results were evaluated visually by overlaying the single-cell mask with the iridium signal (Fig. 2b,c). The image processing steps were chosen such that the final mask was minimally affected by the intensity variation between the slices and that it would yield similar average cell diameters for the *x*–*y* and *x*–*z* directions (Extended Data Fig. 3a,b). Final statistics for each cell were collated in a cell data catalog for easy export and downstream single-cell analysis (Extended Data Fig. 3c). The iridium-channel-dependent segmentation enables the segmentation procedure to be applied to IMC data regardless of the antibody panel.

**Extended Data Fig. 4:**
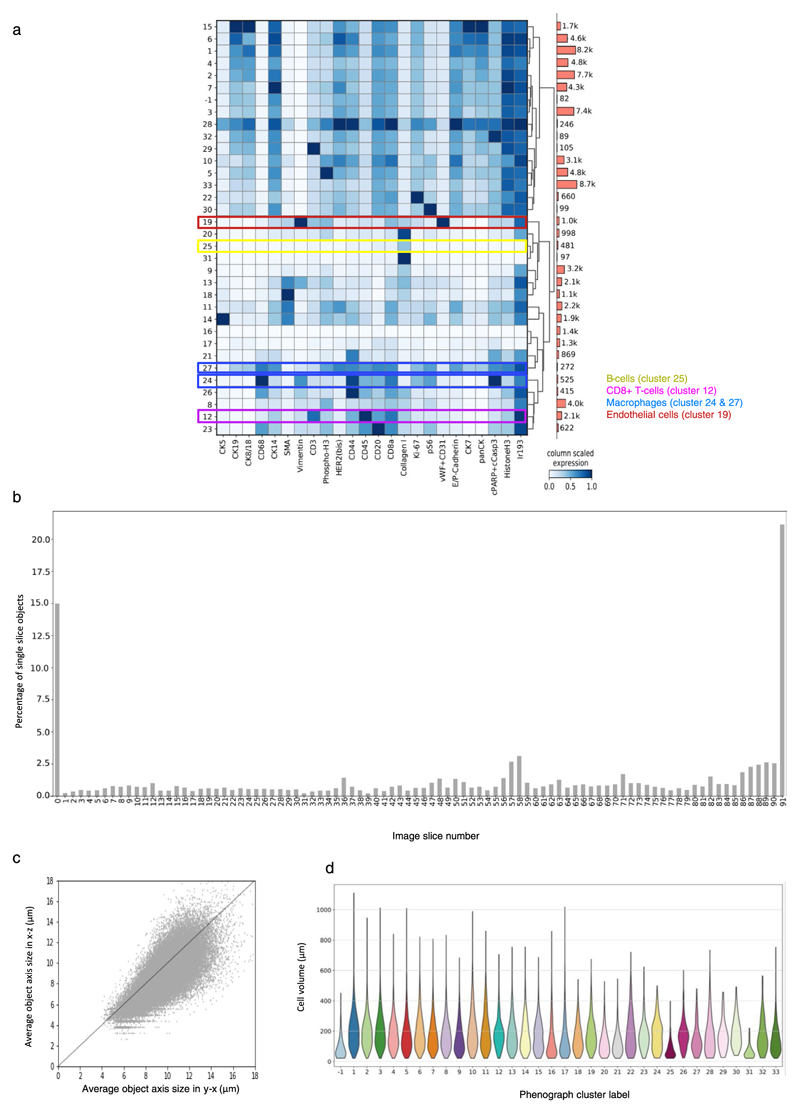
Evaluation of a second breast carcinoma 3D IMC model. (a) Left: Average marker expression (x axis) for each phenotypic cluster (y axis) after measuring the raw data for each segmented cell and clustering cells using Phenograph. Marker expression data for each cell were calculated as the mean intensities of ion counts over the object mask. Cluster –1 are outlier cells identified during clustering. Before clustering, the data was range normalized to the 99^th^ percentile. Right: Bar plot of the absolute cell counts for each cluster. (b) Percentage of objects occurring only on one slice for the full 92-slice stack after 3D segmentation. (c) Average axis length for each object in x-y plane and in y-z plane. The z axis was calculated with 2-μm resolution. Axis length was calculated as the average of minor and major axes using the skimage.mesure.regionprops function. (d) Cell volumes calculated as total number of voxels for each segmented object for each cell cluster identified by Phenograph.

**Fig. 3:**
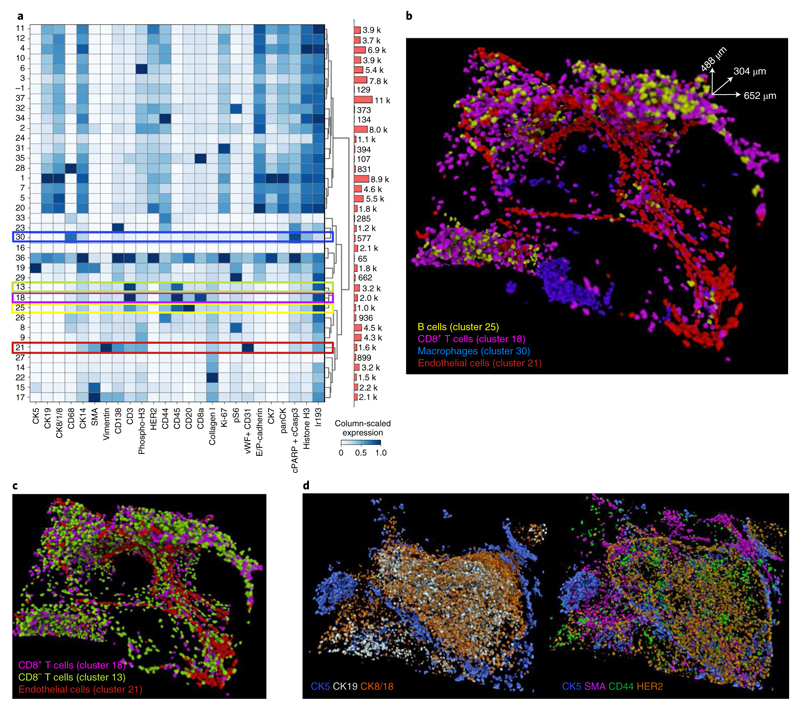
Single-cell analysis of 3D IMC data in a breast carcinoma sample. **a**, Heat map of an average marker expression (*x* axis) for each phenotypic cluster (*y* axis) after measuring the raw data for each segmented cell and clustering the cells using Phenograph from one out of the two breast carcinoma 3D IMC models used in this study (152 slices) (left). Marker expression data for each cell were calculated as the mean intensities of ion counts over the object mask. Colored bars indicate clusters corresponding to specific cell types, color coded as in **c** and **d**. Before clustering, data were range normalized to 99th percentile and z-scored for heat map visualization. Absolute cell counts for each cluster (right). **b**, 3D rendering of cells belonging to cluster 21 (endothelial cells), cluster 18 (CD8a^+^ T cells), cluster 25 (B cells) and cluster 30 (macrophages). **c**, 3D rendering of cells belonging to cluster 21 (vWF^+^CD31^+^), cluster 18 (CD8^+^, CD3^+^ and CD45^+^) and cluster 13 (CD8a^−^, CD3^+^ and CD45^+^). **d**, Single-cell marker expression for CK5, CK19, CK8/18, HER2, CD44 and SMA for each individual cell, where each voxel for every cell is assigned its marker expression value. All renders in this figure are representative images from one of two breast cancer 3D IMC models and are displayed in the same orientation as in Fig. 1b. AGAVE 1.0.0.1 was used for 3D rendering of the data.

To assess whether the derived marker expression for single cells captures expected biological information and aligns with the raw voxel data, we used unsupervised Phenograph clustering to assign a cell phenotype to each single cell (Fig. 3a)^20^. The antibodies in our panel were chosen to enable identification of known tumor, immune, and stromal cell phenotypes. We identified expected cell phenotypes based on differential expression of key markers such as epithelial cells positive for panCK (such as clusters 1–7 and 10–13), stromal cells positive for collagen and SMA (clusters 17 and 22), B cells positive for CD20 and CD45 (cluster 25), T cells positive for CD8a, CD3 and CD45 (cluster 18), macrophages positive for CD68 and CD45 (cluster 30) and endothelial cells positive for von Willebrand factor (vWF) and CD31 (cluster 21). Additionally, the segmented data captured the expected average larger size of tumor cells compared to B cells and T cells (Extended Data Fig. 3d). The segmentation results can be evaluated by overlaying raw voxel data with a cell phenotype mask, where each voxel for every cell is assigned its cluster label (Figs. 1b and 3b and Supplementary Videos 4 and 5). Highlighting the value of 3D data, the location of different cell phenotypes can be easily visualized, for example we observed a clear tendency of CD45^+^CD3^+^ T cells (cluster 13 and 18) to cluster around the vWF^+^CD31^+^ endothelial cells (cluster 21) (Fig. 3c and Supplementary Video 6). Similarly, we observed spatial separation of different subsets of CD68^+^ cells (clusters 30 and 28) (Extended Data Fig. 3e). These distinct patterns would be much more difficult to recognize in 2D. In addition to assigning each cell its phenotype based on aggregated marker expression via clustering, the mean antibody signal statistics for each individual cell can be evaluated together with its spatial location using an expression mask, where each voxel for every cell is assigned the derived marker expression value. We observed that tumor cells in this model showed varying expression levels of luminal cell markers (cytokeratins 8/18 and 19), basal cell markers (cytokeratin 5 and SMA) and other breast cancer epithelial markers such as HER2 and CD44 (Fig. 3d and Supplementary Videos 7 and 8). This shows that 3D IMC captures marker expression heterogeneity in tumor cells as expected from our previous large-scale analyses of breast cancer cohorts^13,21,22^. Reassuringly, applying our pipeline with the same segmentation approach and with the same antibody panel to a second breast carcinoma sample from the same patient yielded similar cell phenotypes (Extended Data Fig. 4a–d and Supplementary Table 3). These analyses demonstrate that 3D IMC enables a detailed analysis of expected cellular phenotypes in a 3D tissue context and identifies cellular populations that differ in location within the tissue volume.

### 3D IMC reveals cellular and microenvironmental relationships

**Fig. 4:**
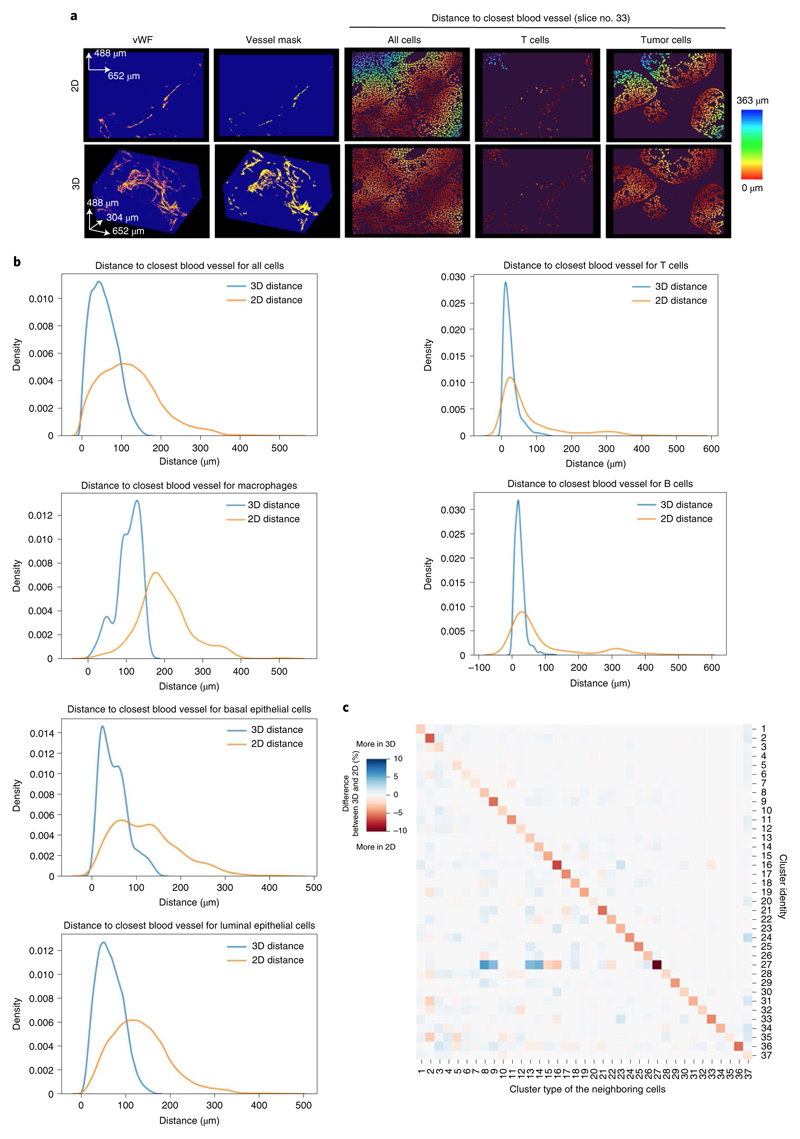
Distance measurement comparison for 3D versus 2D IMC. **a,** Cell-to-vessel distance measurements for the representative slice 33 in the 2D image (top) and 3D reconstruction (bottom) of a breast carcinoma 3D model (as imaged in Figs. 1–3). vWF signals are shown (left to right) to mark vessels, the generated binary masks for vessels, the distance of each cell to the vessel mask in a heat-color scale and distances to the vessel mask of T cells and tumor cells in the same scale. Images were created with napari v.0.4.1rc2 (ref. ^40^). **b,** Quantification of distance from a cell centroid to its closest point in the endothelial cell mask in 2D and 3D for the indicated cell phenotypes in the same model. **c,** Heat map showing the difference between 3D and 2D for the proportion of cluster types among the directly touching neighbors for each cluster across the same breast carcinoma model. For each cell in the model, the total number of times each cluster type was among the neighbors was counted, then these counts were aggregated per cluster by grouping the cells on the basis of cluster assignment. The proportion was calculated by dividing per-cluster counts by total number of neighbors that each cluster had across the whole model. For 2D, the directly touching neighbors were calculated for each slice and averaged cross the whole stack (152 slices). Finally, the difference was calculated by subtracting 2D proportions from 3D proportions.

As tissues are 3D structures, we expected that measurements of distances and intercellular proximity in 3D should better capture the modeled tissue than 2D IMC. We assessed this by comparing various quantitative measures in data obtained from the main HER2-positive ductal breast carcinoma sample described above. We first measured distances between different cell phenotypes, determined with the initial clustering step and the nearest blood vessel in all 2D sections and in the reconstructed 3D model (Fig. 4a). The average distances for different cell phenotypes to the closest blood vessel in 3D were always shorter than when measured in 2D (Fig. 4b). This was as expected, because in single 2D planes the features above and below the plane cannot be detected. To test whether cell–cell interactions differed when analyzed in 3D versus 2D, we determined which cell phenotypes were directly touching for all the cells in each section in 2D and for the entire 3D model. The 3D interaction analysis yielded a different cell interaction picture than 2D analysis. For example, there were more homotypic interactions among all cell types in 2D than in 3D and fewer heterotypic interactions among cluster 25 (B cells) and cluster 18 (T cells) in 2D (Fig. 4c). Plausible explanations for these differences are under-sampling in the 2D space and the existence of interactions that occur in constrained directions (such as lymphocytes tethered to blood vessels). These two analyses suggest that a 3D model yields quantitatively different measures of cellular and microenvironmental relationships than a 2D image and that the differences are expected or plausible given the 3D tissue architecture.

**Fig. 5:**
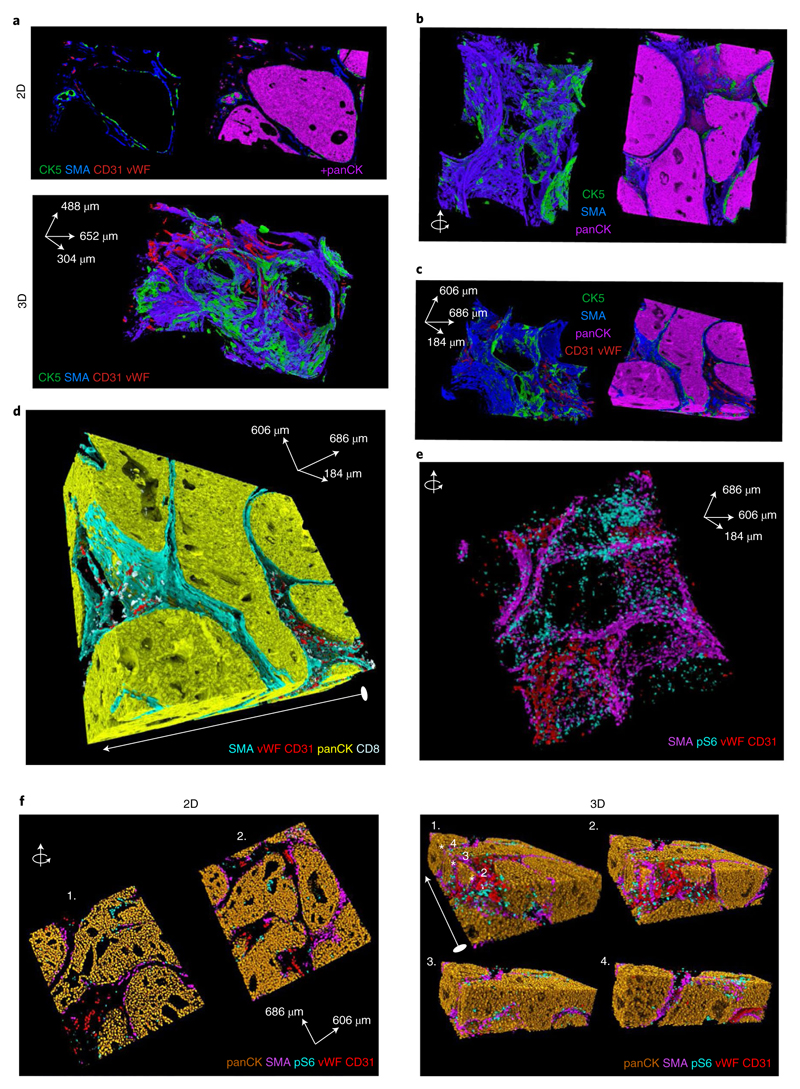
3D reconstructions reveal cellular and environmental relationships in tissues. **a**, Raw data rendering in 2D and 3D of a tumor basal layer, showing expression of basal markers CK5 and SMA, and endothelial markers vWF and CD31 in one out of the two breast carcinoma 3D IMC models used in this study. The 2D slice is representative of 152 slices; the 3D rendering uses all of the 152 slices. The image has the same angle as images in Fig. 3b–d. **b**, Alternative angle of the same model showing raw data rendering of the indicated markers. **c**, Raw data voxel rendering of the second breast cancer 3D IMC model showing SMA, vWF, CD31, panCK and CD8a marker distributions. The white arrow indicates the angle of the model displayed on **d** and **e**. **d**, Raw data voxel renderings of the same breast carcinoma 3D IMC model as in **c** showing SMA, panCK, vWF, CD31, and CK5 marker distributions. **e**,**f**, Single-cell expression for the indicated markers in the same breast carcinoma model shown in **c**. Each voxel for every cell is assigned an expression value for each marker. Two representative 2D slices (out of 92) from the breast carcinoma model imaged in **c** and **d**, showing single-cell level expression for the indicated markers (**f**, left). Single-cell expression for panCK, SMA, vWF, CD31 and pS6 shown in a full 3D model for the breast carcinoma model analyzed in **e** and **f**^1^ and displayed through the z-dimensions cut at different *x*-planes of the model^2–4^ (**f**, right). The white arrow indicates the direction along which the model was cut and the white stars indicate the positions of the cuts. AGAVE 1.0.0.1 was used for 3D rendering of the data.

We also reasoned that 3D models would reveal spatial arrangements of protein expression that might not be visible on 2D images. In our main HER2^+^ ductal breast carcinoma 3D model, we observed a panCK^+^ luminal region and a patchy and discontinuous pattern of cytokeratin 5 (CK5) in the basal layer, although SMA, another basal marker, had uniform expression (Fig. 5a,b). The patchy basal layer is only clearly visible in the 3D reconstruction rather than in the 2D image (Fig. 5a,b and Supplementary Video 9). We observed a similar discontinuous pattern of CK5 in the second breast carcinoma sample reconstructed from the same HER2^+^ ductal breast patient sample using the 3D IMC workflow (Fig. 5c). The second model is composed of large epithelial tumor compartments and a stromal compartment with a CD8a^+^ region (Fig. 5d and Supplementary Video 10). Understanding these different phenotypes in the basal layer may shed light on the early steps of invasion in ductal carcinomas. Additionally, with cell-level marker expression data in the second breast carcinoma model, we observed in 3D that pS6^+^ cells line the epithelial tumor compartments (Fig. 5e). In 2D, the distribution of pS6^+^ cells seems disordered and the distance to the SMA^+^ cell area is unclear (Fig. 5f). In the 3D reconstruction, however, it is apparent that pS6^+^ cells colocalize with SMA^+^ cells (Fig. 5f and Supplementary Video 11). These examples illustrate that spatial cell-level organization become apparent in 3D, underscoring the value of our approach for studying tissue architecture.

### Spatial invasion-associated events captured by 3D models

**Extended Data Fig. 5:**
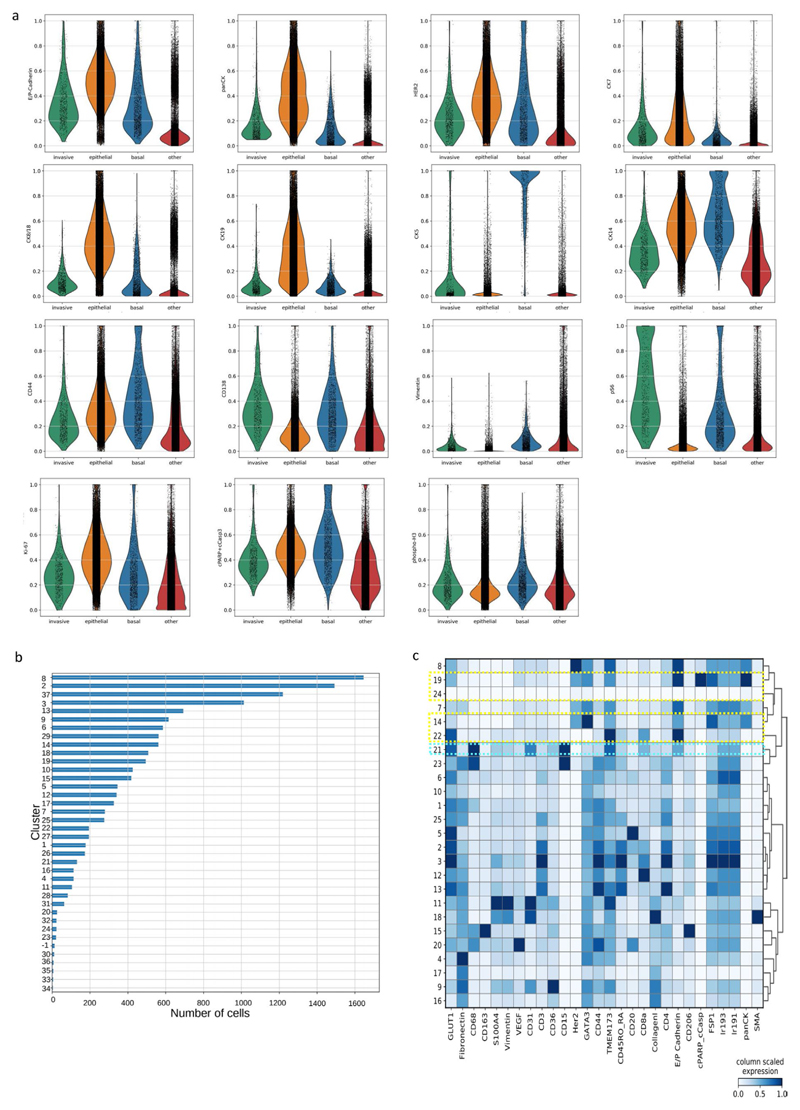
Analysis of putative invasive cells and single-cell level data for a breast cancer lymphovascular invasion model. (a) Violin plots showing marker expression levels for putative panCK+ invasive cells compared to epithelial, basal and other cells in a HER2-positive ductal breast carcinoma model. (b) A bar plot showing the total number of cells from each cluster (Fig. 2b) in the immediate microenvironment of putative invasive cells in the breast carcinoma model. Microenvironment was defined as cells that are within 50 μm radius from any of the putative invasive cells. (c) Analysis of segmented cell-level data in a lymph vessel lymphovascular invasion model. Average marker expression (x axis) for each phenotypic cluster (y axis) after measuring the raw data for each segmented cell and clustering cells using Phenograph. Marker expression data for each cell were calculated as the mean intensities of ion counts over the object mask.

**Fig. 6:**
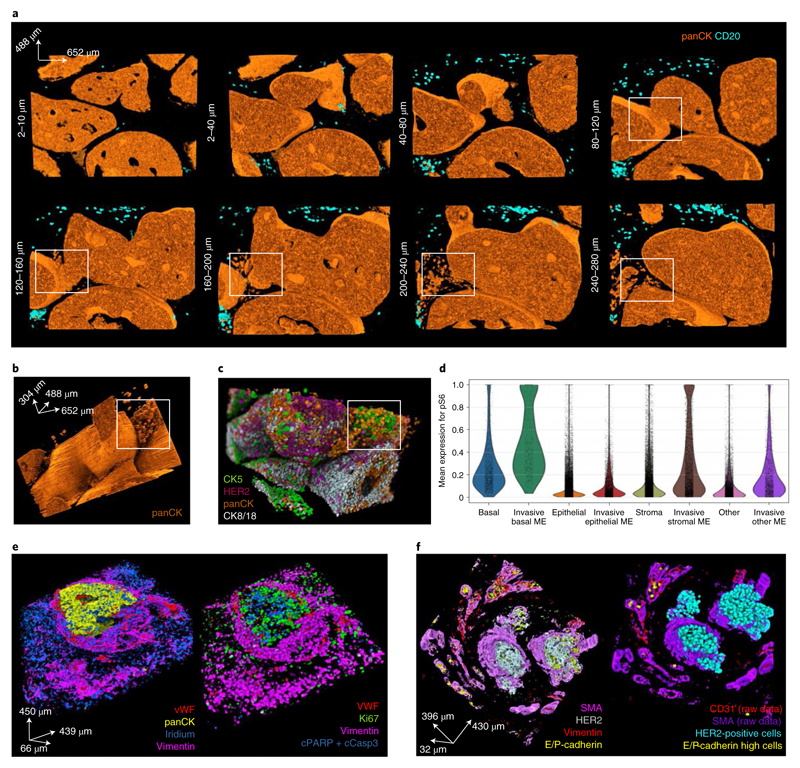
3D IMC reveals potential invasion-associated phenomena. **a**, A series of raw voxel data 3D renderings of ~40 μm stacks (20 slices) for panCK and CD20 from the breast carcinoma 3D IMC model that captured putative invasive cells (same model as displayed in Fig. 1). The white rectangle marks a protrusion in the surface of a ductal carcinoma in situ-like structure at 120 μm depth and marks tumor clusters at the same location. **b**, Full 3D raw data voxel rendering of panCK from the same model as **a**. The tumor cell clusters are shown within a white rectangle. **c**, Single-cell marker expression levels for CK5, panCK, HER2 and CK8/18 visualized by rendering over the 3D mask. Marker expression data for each cell was calculated as the mean intensity of ion counts over the object mask. **d**, A violin plot for pS6 expression levels in epithelial, basal and stromal cells in the immediate microenvironment (ME) of invasive cells and in cells of the same type in the whole tissue. ME was defined as cells that are within 50-μm radius from any of the putative invasive cells. **e**, A rendering of a raw data 3D IMC voxel model from one out of two pathologist-identified samples of lymphovascular invasion taken from the periphery of a breast tumors (left). Single-cell marker expression for the same model where each voxel for every cell is assigned its marker expression value for the indicated markers, including proliferating tumor cells (Ki-67) and apoptotic cells (positive for cleaved PARP and cleaved caspase 3) (right). **f**, Raw data 3D IMC voxel model of a pathologist-identified sample of lymphovascular invasion from a different breast tumor, out of the two such samples used in this study (left). Single-cell marker expression for the same model showing HER2^+^ tumor cells inside a lymph vessel in the center of the image and single E-cadherin^+^ cells inside blood vessels at the periphery of the image (right). AGAVE 1.0.0.1 was used for 3D rendering of the data.

3D reconstructions could be particularly powerful in analysis of phenomena that proceed through 3D space. Reconstruction of the main HER2^+^ ductal breast carcinoma 3D model revealed a structure that might resembles a microinvasive lesion and a stream of potentially invasive tumor cells (Fig. 6a). The distal part of the tumor (up to a depth of 80 μm along the *z* axis) contained no putative invading cells and the tumor cell compartments had smooth tumor–stroma boundaries (Fig. 6a). Toward the center of the tumor (about a depth of 120 μm along the z axis) and through the proximal area (160 μm and deeper), putative invasive panCK^+^ epithelial cells potentially emerging from a microprotrusion were detected (Fig. 6a,b and Supplementary Video 12). In the 3D model, these potentially invasive cells could be differentiated from the bulk tumor mass with confidence, as it is possible to determine whether a given cluster of epithelial cells is completely disconnected from the tumor network or not. This is impossible in a single or few 2D sections. We observed that these cells differed from the tumor bulk in the expression level for key epithelial cell markers (Fig. 6c and Supplementary Video 13). The putative invasive cells had lower expression of epithelial markers such as CK8/18, CK19 and HER2 and higher expression of CD138 and pS6 than other epithelial cells in the whole model (Extended Data Fig. 5a). We also observed that CK5^+^ basal cells and stromal cells in the immediate microenvironment (50-μm radius from each putative invasive cell) also had increased levels of pS6 expression compared to other CK5^+^ basal and stromal cells in this model (Fig. 6d and Extended Data Fig. 5b).

We reconstructed two additional 3D models to further illustrate the capabilities of 3D IMC for analyzing spatially confined events. These samples were identified by pathologists as lymphovascular invasion sites at the periphery of two different HER2^+^ breast carcinomas and as such should capture metastasis-associated events. Presence of lymphovascular invasion in breast cancer has been associated with worse patient outcome^23^ and various genes have been associated with a higher likelihood of the event^24,25^, but they are challenging to analyze on account of their 3D topology. Multiplexed 3D IMC enabled us to evaluate cell subsets and their 3D spatial distribution in such lymphovascular clusters. For instance, in one of the models (Fig. 6e, Supplementary Video 14 and Supplementary Table 4), a large panCK^+^ tumor cell cluster is visible inside a blood vessel that is positive for vWF, CD31 and vimentin. Similarly, we captured a HER2^+^ and E/P-cadherin^+^ tumor cell cluster inside a lymph vessel (SMA^+^, CD31^−^ and vimentin^−^) and single E-cadherin^+^ cells in the surrounding blood vessels (positive for CD31 and vimentin) (Fig. 6f, Supplementary Video 15 and Supplementary Table 4), in the second lymphovascular invasion model. Clustering-based phenotypic analysis revealed higher expression of HER2 and lower expression of TMEM173 in the large lymph vessel-invaded cluster compared to the single blood vessel-invaded cells (Extended Data Fig. 5c). Future analysis could involve an analysis of cell phenotypes inside the lymphovascular clusters in comparison to the whole tumor mass. Confident assignment of the properties of potentially invasive tumor cell clusters and their associations with metastases will require further study, but these three examples highlight the suitability of multiplexed 3D IMC for studying spatial events such as cell invasion during metastasis.

## Conclusions

Here we demonstrate a multiplexed, single-cell resolution, 3D analysis strategy for FFPE tissues. 3D IMC will enable detailed visualization and analysis of cell lineage and communication mechanisms in the context of native tissue architecture for any tissue type. Studies using multiplexed 2D imaging methods have shown a correlation between spatial cell communities and clinical outcomes in breast and colorectal cancer^21,26,27^ and difference in cell interaction patterns has been linked to disease progression in diabetes^28^. We have demonstrated that measurements of the spatial neighborhood can differ between 3D and 2D images. As 3D measurements are expected to better capture tissue composition compared to 2D representation, 3D IMC could prove especially beneficial for studying the effect of microenvironment on tumor cell biology. Additionally, 3D methods have been indispensable for studying tumor invasion into blood vessels in pancreatic cancer patient samples^29^ or identifying tumor budding in samples from patients with pancreatic, colorectal, lung and breast cancer^30^ because neither of the phenomena can be represented clearly in a single 2D image. 3D IMC would be suitable for capturing such events and thanks to the multiplexed nature of IMC, our method would facilitate a detailed understanding of cell characteristics associated with invasive events.

Additional computational and technical developments will further improve the utility of 3D IMC. For instance, acquisition time is long (4 full days for each tumor sample). Development of hardware with higher ablation speed will allow shorter acquisition times or, alternatively, better resolution. As the amount of 3D IMC data increases, better 3D cell segmentation approaches based on machine learning methods could also be implemented^31^, which could provide more accurate cell shape representations and reduce unclear cell phenotypes. Furthermore, newly developed visualization and analysis tools, including virtual reality applications, to augment the amount of data that can be simultaneously visualized in 3D^32,33^, will further facilitate analysis of such detailed 3D datasets. With our accessible computational pipeline based on publicly available tools and a growing set of well-validated antibodies among IMC users, we envision that 3D IMC could be increasingly employed by different laboratories. In conclusion, the 3D IMC approach advances the study of the spatial distribution of proteins in the context of 3D tissue architecture.

## Methods

### Statement of research integrity

This study complies with all relevant ethical regulations and was approved by the ethics committee of the Canton Zurich (PB_2016-00811).

### Breast cancer tissue samples

We used archived material from the tissue biobank at the University Hospital Zurich. Regions of interest in different breast carcinoma samples were identified in hematoxylin and eosin-stained sections from FFPE tissue blocks by a pathologist and 1-mm or 1.5-mm diameter cylindrical punches were obtained. The main and the second breast carcinoma 3D models were generated from one patient sample. The blood and the lymph vessel lymphovascular invasion models were generated from two different patient samples. Another sample from a patient with breast carcinoma was used for comparing different antigen retrieval conditions

### Antibody testing and validation

Antibody panels were designed using AirLab^34^. All antibodies (Supplementary Tables 2–4 and Extended Data Fig. 1b) were conjugated to metals using the MaxPar X8 Multimetal Labeling Kit (Fluidigm) according to the manufacturer’s instructions. Before testing antibodies, manufacturer’s website and antibody databases were used for choosing antibodies for targets of interest. Antibodies were initially tested using immunofluorescence staining without metal conjugation in lymph node, spleen and breast cancer FFPE sections. Antibodies that revealed expression patterns consistent with the literature were chosen for metal conjugation. After the conjugation, another round of testing was undertaken using IMC with breast cancer FFPE sections and antibodies that showed staining patterns consistent with the literature and with sufficient signal intensity were utilized. In this step, thanks to multiplexing capabilities of IMC, often already validated antibodies were used in addition to the antibodies being tested to validate cell type specific staining (epithelial and different immune cells). Breast cancer FFPE tissues were used for titrating antibodies for final IMC measurements.

### Sample sectioning with ultramicrotome

For generating a 3D IMC model, first a tissue punch from an FFPE sample was re-cast into an EPON silicon mold by first putting the sample into a container of molten paraffin for a few minutes to clear the remaining paraffin and then placing the tissue into the silicon mold with the cylinder axis along the axis of the mold. After which, the mold was filled with fresh paraffin and placed at 4 °C. Once the paraffin was solidified (about 1 h), a tissue block was mounted in an ultramicrotome capsule holder and a diamond trimming knife was used to trim a trapezium shape with tissue exposed in the salient block face. Trimming was required to enable adherence of consecutive sections into ribbons that then could be collected on a microscopy slide. A Histo-Jumbo Diamond knife (Diatome) with a plastic-sealed hole in the bottom was used for cutting sections. A needle connected to a syringe filled with ultrapure water was inserted through the bottom hole, a Superfrost histological glass slide was placed in a boat of the knife and then the knife boat was filled with ultrapure water until the water was level with the knife edge. With step length set to 2 μm, consecutive slices were cut. The water level in the boat was reduced using the syringe to collect ribbons on the microscopy slides. These slides were then dried on a warm metal plate (30 °C) for 2 h or left overnight at room temperature to tether tissues to the glass slide.

### Tissue preparation and antigen retrieval

Tissue sections were dewaxed in xylene for at least 2 h and again in fresh xylene at least 1 h and then in xylene:ethanol (1:1) for 10 min. Samples were rehydrated in a graded series of alcohol (ethanol:deionized water 100:0, 90:10, 80:20, 70:30, 50:50 and 0:100; 10 min each). After that, slides were put into to Tris-EDTA buffer at pH 9.2 followed by heat-induced epitope retrieval in a pressure cooker (Medite) for 30 min at 95 °C or for 80 min at 80 °C. Samples were left to cool in the Tris-EDTA buffer for 20 min followed by cooling in Tris-buffered saline (TBS) at room temperature for at least 20 min and then blocked with blocking buffer containing 3% BSA in TBS or TBS with 1% horse serum and 1% FcBlock (Miltenyi Biotech) for about 1 h. Samples were then stained with antibody mix (diluted in blocking buffer) and incubated overnight at 4 °C. Samples were washed three times in TBS for 5–10 min each wash before incubating for 2–5 min with iridium intercalator diluted in TBS followed by three washes in TBS (5 min each wash) or by a 10-s wash in PBS if ruthenium staining was used in the next step. For ruthenium counterstaining^15^, ruthenium tetroxide (0.5% solution from Electron Microscopy Sciences) was diluted 1:1,000 in PBS. Samples were incubated for 3–5 min and then samples were washed in ultrapure water. Samples were then air dried before IMC acquisition. All samples were subjected to these steps with the following differences: the samples for the main and the second breast carcinoma model were subjected to HIER antigen retrieval for 80 min at 80 °C and were counterstained with ruthenium; the lymphovascular invasion models were subjected to HIER for 30 min at 95 °C without the ruthenium counterstaining step.

### Imaging mass cytometry

Data acquisition was performed on a Helios time-of-flight mass cytometer (CyTOF) coupled to a Hyperion Imaging System (Fluidigm). Selected areas for ablation were larger than the actual area of interest to account for loss of overlapping areas among sections due to cumulative rotation.

The selected area ablated per section was around 1 mm^2^. Laser ablation was performed at a resolution of approximately 1 μm with a frequency of 200 Hz (or 400 Hz for lymphovascular invasion models) with estimated acquisition time of 1 mm^2^ h^−1^. To ensure performance stability, the machine was calibrated daily with a tuning slide spiked with five metal elements (Fluidigm). All data were collected using the commercial Fluidigm CyTOF software v.01.

### Data processing

MCD files from the Fluidigm Hyperion imaging system were converted into OME-TIFFs using publicly available IMC data preprocessing pipeline (https://github.com/BodenmillerGroup/ImcSegmentationPipeline) based on imctools v.1.0. OME-TIFFs were then converted to single-channel 16-bit TIFF stacks and if necessary zero-padding was added to make the images the same size across a 3D stack. For the main breast carcinoma model, some images that were acquired in multiple acquisition attempts required reassembly. The 152 slices analyzed corresponded to 168 IMC acquisitions with 10 cases where a section was contained in two or three different IMC images. For the main breast cancer model slice 16 is missing and for the second breast carcinoma model slice 59 is missing; missing slices were omitted from downstream analysis.

### Image registration

The following Fiji-ImageJ2-linux64 v.1.0 (ref. ^35^) plugins accessed via Jython scripting were used for image registration: Register Virtual Stack Slices was used to automatically extract SIFT features to calculate a transformation matrix based on a chosen registration model and Transform Virtual Stack Slices was applied to align images from all the channels (https://github.com/fiji/register_virtual_stack_slices/). For an image stack alignment for any 3D IMC model, first a single-channel stack containing images stained for smooth muscle acting were histogram equalized per image and using Register Virtual Stack Slices plugin a similarity (translation + rotation + isotropic scaling) model was applied to this stack to find a transformation matrix for the alignment. The Register Virtual Stack Slices plugin was run with the following default parameters: maxOctaveSize = 1,200, minInlierRatio = 0.05 (RANSAC true matches ratio), featuresModelIndex = RIGID, interpolate = true, rod = 0.86 (closest neighbor distance ratio). The parameter for maxEpsilon = 10 (maximal allowed alignment error in pixels) was determined empirically by calculating a cross-correlation for a small region in the middle of the aligned iridium channel image stack and choosing the parameter with highest cross-correlation value. Once a transformation matrix was determined, then the Transform Virtual Stack Slices plugin was used for separate image stacks for each of the channels present in the antibody panel. Finding the best alignment approach and applying the transformations to all the channels takes about 2 h depending on the number of slices in the model.

### Single-cell segmentation

After aligning images across all channels, the stack of raw iridium images was used as a starting point for the input to 3D watershed algorithm^36^. Segmentation using the nuclear channel facilitates the independence of this pipeline from the antibody panel as antibody panels differ between models. All the steps for segmentation were performed in Fiji-ImageJ2-linux64 v.1.0 (ref. ^35^) unless custom Python scripts relying heavily on the NumPy package^37^ are specified. The segmentation pipeline involved multiple commonly used digital image processing steps as described below. The different steps and parameters used were determined empirically by visually assessing the overlap between the cell mask and the nuclear iridium signal (Extended Data Fig. 3b,c). The following steps resulted in single-cell segmentation:

1. Mean artificial images were generated, where each artificial image was calculated using custom Python script as the mean of iridium channel between a preceding and a subsequent image resulting in matching *x*–*y*–*z* pixel resolution of 1 μm. The image stack composed of measured and artificial images was then used for all the downstream segmentation steps, but not for single-cell data measurements.
2. ¾e raw iridium channel intensity counts for 16-bit images were clipped to value 50 to remove bright outlier pixels followed by histogram equalization using the square root of histogram values with 0.3% noise parameter to enhance details of the image without extreme effects of classical histogram equalization. As histogram equalization enhances noise, the background pixels were removed. The minimum cutoff value was determined by eye for each model separately (for the main breast carcinoma model, the cutoff was 11,467; for the second breast carcinoma model it was 14,862; for the first lymphovascular invasion model (with blood-vessel-invaded tumor cell cluster) it was 20,243 and for the second lymphovascular invasion model (with lymph-vessel-invaded tumor cell cluster) it was 33,760).
3. Due to varying intensity of iridium staining on some of the images, further pixel normalization across each image was required before implementation of the watershed algorithm. CLAHE, a local histogram equalization algorithm that enhances local contrast^38^), was applied (parameter block size = 29, maximum slope 10,256 bins, fast = false). Subsequently, local contrast enhancement was applied that used means and standard deviations of local image regions to normalize pixel values (https://github.com/axtimwalde/mpicbg/blob/master/mpicbg/src/main/java/mpicbg/ij/integral/NormalizeLocalContrast.java); the block size was 10 and s.d. sigma value was 3.
4. After pixel value normalization, a median 3D filter with kernel size of 3 × 3 × 3 was followed by 3D Gaussian blur (sigma = 1) to integrate pixel values across the 3D stack.
5. The difference of Gaussians for each image in the stack (between sigma 2 and sigma 3) was used as an input to the watershed segmentation algorithm. This approach was chosen as the difference of Gaussian filters is commonly used for blob detection algorithms including nuclei segmentation.
6. An implementation of watershed called h-watershed (available from https://github.com/mpicbg-scicomp/Interactive-H-Watershed/) without interactive window was used for 3D nuclear segmentation (background parameter = 500 for 16-bit images and 90% flooding threshold). The output from this step was a stack of cell masks equivalent to the input stack where each object is marked with a unique identifier.
7. Images containing cell masks for the artificial mean images were removed and only masks for true images were kept for next steps, including the final single-cell measurements.
8. Further post-processing of the segmentation mask obtained from h-watershed was performed using Python scikit-image 1.5.2 package^39^. The following steps were implemented: (1) objects with size smaller or equal to 10 voxels were removed; (2) for rare objects that were disconnected on an *x*–*y* plane but connected in *z* plane, the smaller part of the object was removed; (3) all remaining object labels were expanded by 1 pixel on *x*–*y* plane; (4) objects smaller than 21 voxels were removed; and (5) objects with raw mean iridium signal <1 were removed.
9. This final cell mask was then cut so that only the overlapping area for the whole 3D stack was analyzed. Dimensions for choosing the overlapping area were determined by eye and differed for each model.

Image preprocessing for this segmentation pipeline takes about 30 min. The 3D watershed implementation takes a few minutes for smaller models (16–30 slices) and a few hours for larger models (92–152 slices). Post-processing of the segmentation mask takes about 30 min. This segmentation workflow greatly benefited from a visualization tool easily launched from Jupyter notebooks via Python called napari v.0.4.1rc2 (ref. ^40^).

### Single-cell data measurements

The final single-cell masks obtained from the segmentation pipeline were used to measure raw data for each marker for each cell. Mean intensity for each channel was measured as implemented in the skimage. measure.regionprops function. Before measuring mean intensity per object, hot pixels caused by artifacts of antibody staining were removed for each channel image stack analogously to our standard 2D IMC segmentation pipeline based on CellProfiler plugins. The hot pixels were given the value of the maximum local neighbor intensity (kernel size 3 × 3). Hot pixels were classified as any pixels that are 50 units higher than the difference between the raw data image and local maximum intensity. After measuring the mean intensities for each object for each channel, the mean intensities were compensated to reduce the rare ‘spillover’ between metal isotopes using the compensation matrix described previously^41^. The final output from this step was exported as a csv matrix for downstream single-cell analysis.

### Single-cell clustering

Single-cell marker expression data was clustered with Python3 implementation of Phenograph^20^ (https://github.com/jacoblevine/PhenoGraph) using Manhattan distance and ten nearest neighbors. Before clustering, marker expression was range normalized to the 99th percentile across all cells for each channel separately to give equal weight to each marker. The z-scored cluster means were displayed as heat map using the Python Scanpy package^42^.

### Assessment of single-cell segmentation results

The following multipronged strategy was used for assessing the performance of the segmentation approach. (1) Visually assess whether individual cell masks overlapped with direct measurements of a nuclear signal stained with iridium and whether individual cell masks were separated if clear separation was visible in the nuclear signal. (2) Visually assess overlap between raw voxel-level data and the final segmentation masks for expected cell types, such as epithelial cells in blood vessels and immune cells (Supplementary Video 4). (3) Check that the final segmentation mask is not affected by intensity variation between the sequential slices in the rare cases where such variation occurs that most segmented objects extend over multiple slices as expected by calculating the number of objects that are only present on one slice for each slice in the model (except on the first and last slice of the image deck) (Extended Data Figs. 3a and 4b). (4) Check that the 3D watershed algorithm uses information from *z* direction and the final objects are not biased in *x*–*y* direction compared to *z* direction by measuring the average axis length in *x*–*y* direction and *x*–*z* direction for each object present in the model (Extended Data Figs. 3b and 4c). (5) Check whether the resulting cell volume for cells belonging to different phenotypic clusters captured the expected average larger size of tumor cell phenotypes in comparison to immune cell phenotypes (Extended Data Figs. 3d and 4d). (6) Assess whether the unsupervised clustering method of the resulting segmented data yielded the expected phenotypes based on the antibody panel, such as CD20^+^ B cells, CD8^+^ T cells, CD68^+^ macrophages, vWF^+^CD31^+^ endothelial cells, CK5^+^ basal cells and different clusters of cytokeratin-positive cells in accordance with biological knowledge of the respective marker expression of these cell types. The clustering method also separated a group of rare objects that are high for all the markers due to experimental artifacts such as folds in the paraffin.

### Distance measurements to closest blood vessel

First, the center of each segmented cell in 3D or separately for each slice in the model for 2D analysis was determined by using skimage.measure.regionprops.centroid function in Python using the single-cell mask TIFF stack the main Her2^+^ breast carcinoma model. For 2D analysis, the 3D segmentation mask for each individual 2D slice was used. Second, blood vessel mask was created with Fiji-ImageJ2-linux64 v.1.0 using an image of vWF+CD31 channel by first normalizing the 16 bit raw data TIFF image, then applying Gaussian blur (sigma 1) and using Renjyi entropy filter to generate a binary vessel mask image. Third, for each cell centroid the Euclidean distance to the closest blood vessel mask pixel was calculated. Because in the 3D single-cell mask TIFF stack objects occur over several slices, for the 2D calculation the minimum distance over all the slices that a cell occurs was taken as the final distance measurement. All the measurements for each cell were then average over phenotypic clusters determined in the previous step.

### Measurement of direct neighbors

Direct touching neighbors were measured for the main Her2^+^ breast carcinoma model using skimage region adjacency graph function for each object in the whole 3D model and in each individual 2D slice using the single-cell mask TIFF stack. For 2D analysis, the final 3D segmentation mask for each individual 2D slice was used. For each cell the proportion of neighboring cells belonging to each phenotypic cluster (determined earlier with Phenograph clustering) was determined in 3D or for each 2D slice using a respective region adjacency graph. First, for each cell the number of cells belonging to each cluster among its directly touching neighbors was counted. Second, from the previous step, all the cells were separated based on the cell type (cluster) assignment and then for each cell type (cluster), the number of cells belonging to each of the cluster was counted (for example, cell type 1 (cluster 1) had a cell type 2 (cluster 2) as a directly touching neighbor in the model for 500 times). Third, for each cell type (cluster) the total number of neighbors was calculated by summing the cell counts across the different clusters and then the proportions were determined by dividing the per-cluster cell counts (step 2) by the total number of neighbors. These steps were repeated for each individual 2D slice. Finally, the difference in the 3D and 2D proportions was calculated by subtracting 2D proportions from 3D proportions.

### Analysis of putative invasive cells

The putative invasive cells were determined in the main Her2^+^ breast carcinoma model by manually selecting and recording the cell label for cells that were detached from the main tumor bulk and were present in the putative invasive region and had mean expression of pan-cytokeratin >0.2 (the expression levels were range normalized to 99th percentile across all the cells for each marker in the antibody panel) using napari v.0.4.1rc2 (ref. ^40^). A total of 763 putative invasive cells were found. The microenvironment was determined by finding the cell label for all other cells that were within 50-pixel radius from the centroid of each invasive cell. Cell phenotypes were based on the Phenograph clustering conducted in the previous step. A cell was considered to be epithelial if it belonged to clusters 1–7, 11, 20, 23, 24, 31–35 or 37; basal if it belonged to cluster 19, stromal if it belonged to clusters 8–10, 12, 14, 15, 17, 22, 26, 27 or 29; or ‘other’ in all remaining cases.

### 3D data visualization

Agave v.1.0.0.1 (https://www.allencell.org/software-and-code.html) was used for 3D voxel or object rendering. Before voxel rendering 16-bit TIFF stacks were created for channels of interest. Each image was first histogram equalized, then background pixels were removed and a Gaussian blur filter with radius 1 was applied. Fiji-ImageJ2-linux64 v.1.0 was used to create image stacks for Agave rendering. Supplementary Videos 2–14 were created using Agave v.1.0.0.1 by taking series of images after rotating the model a fixed degree and then assembling the images into an animation.

### Statistics and reproducibility

No statistical method was used to predetermine sample size; however, reproducibility of the method has been demonstrated on four different samples. No data were excluded from the analyses. The experiments were not randomized. The Investigators were not blinded to allocation during experiments and outcome assessment^15,20,34–42^.

### Reporting Summary

Further information on research design is available in the Nature Research Reporting Summary linked to this article.

## Extended Data

## Supplementary Material

Reporting Summary

Sup_Vdo_1

Sup_Vdo_2

Sup_Vdo_3

Sup_Vdo_4

Sup_Vdo_5

Sup_Vdo_6

Sup_Vdo_7

Sup_Vdo_8

Sup_Vdo_9

Sup_Vdo_10

Sup_Vdo_11

Sup_Vdo_12

Sup_Vdo_13

Sup_Vdo_14

Sup_Vdo_15

Supplementary Information

Supplementary tables

## Data Availability

All the data required for the 3D models from this study including IMC high-dimensional tiff images, single-cell masks and single-cell data are available at https://doi.org/10.5281/zenodo.4752030. Source data have been provided as Source Data files. All other data supporting the findings of this study are available from the corresponding author on reasonable request.

## Code availability

All code that was used for preprocessing images and analyzing single-cell data is available at https://github.com/BodenmillerGroup/3D_IMC_publication.

## References

[1] Coutu D, Kokkaliaris K, Kunz L, Schroeder T (2018). Multicolor quantitative confocal imaging cytometry. Nat Methods.

[2] Li W, Germain RN, Gerner MY (2017). Multiplex, quantitative cellular analysis in large tissue volumes with clearing-enhanced 3D microscopy (Ce3D). Proc Natl Acad Sci USA.

[3] Mano T (2018). Whole-brain analysis of cells and circuits by tissue clearing and light-sheet microscopy. J Neurosci.

[4] Gerdes MJ (2013). Highly multiplexed single-cell analysis of formalin-fixed, paraffin-embedded cancer tissue. Proc Natl Acad Sci USA.

[5] Gerdes MJ (2018). Single-cell heterogeneity in ductal carcinoma in situ of breast. Mod Pathol.

[6] Goltsev Y (2018). Deep profiling of mouse splenic architecture with CODEX multiplexed imaging. Cell.

[7] Schubert W (2006). Analyzing proteome topology and function by automated multidimensional fluorescence microscopy. Nat Biotechnol.

[8] Murray E (2015). Simple, scalable proteomic imaging for high-dimensional profiling of intact systems. Cell.

[9] Wang X (2018). Three-dimensional intact-tissue sequencing of single-cell transcriptional states. Science.

[10] Angelo M (2014). Multiplexed ion beam imaging of human breast tumors. Nat Med.

[11] Thiele H (2014). 2D and 3D MALDI-imaging: conceptual strategies for visualization and data mining. 2D and 3D MALDI-imaging: conceptual strategies for visualization and data mining. Biochim Biophys Acta Proteins Proteom.

[12] Coskun AF (2021). Nanoscopic subcellular imaging enabled by ion beam tomography. Nat Commun.

[13] Giesen C (2014). Highly multiplexed imaging of tumor tissues with subcellular resolution by mass cytometry. Nat Methods.

[14] Schulz D (2018). Simultaneous multiplexed imaging of mRNA and proteins with subcellular resolution in breast cancer tissue samples by mass cytometry. Cell Syst.

[15] Catena R, Montuenga LM, Bodenmiller B (2018). Ruthenium counterstaining for imaging mass cytometry. J Pathol.

[16] Chang Q, Ornatsky O, Hedley D (2017). Staining of frozen and formalin-fixed, paraffin-embedded tissues with metal-labeled antibodies for imaging mass cytometry analysis. Curr Protoc Cytom.

[17] Bouzekri A, Esch A, Ornatsky O (2019). Multidimensional profiling of drug-treated cells by imaging mass cytometry. FEBS Open Bio.

[18] Schapiro D (2017). histoCAT: analysis of cell phenotypes and interactions in multiplex image cytometry data. Nat Methods.

[19] Sy S, Ang LC (2019). Microtomy: cutting formalin-fixed, paraffin-embedded sections. Biobanking.

[20] Levine JH (2015). Data-driven phenotypic dissection of AML reveals progenitor-like cells that correlate with prognosis. Cell.

[21] Jackson HW (2020). The single-cell pathology landscape of breast cancer. Nature.

[22] Raza Ali H (2020). Imaging mass cytometry and multiplatform genomics define the phenogenomic landscape of breast cancer. Nat Cancer.

[23] Song YJ (2011). The role of lymphovascular invasion as a prognostic factor in patients with lymph node-positive operable invasive breast cancer. J Breast Cancer.

[24] Klahan S (2017). Identification of genes and pathways related to lymphovascular invasion in breast cancer patients: a bioinformatics analysis of gene expression profiles. Tumor Biol.

[25] Kurozumi S (2019). A key genomic subtype associated with lymphovascular invasion in invasive breast cancer. Br J Cancer.

[26] Schürch CM (2020). Coordinated cellular neighborhoods orchestrate antitumoral immunity at the colorectal cancer invasive front. Cell.

[27] Keren L (2018). A structured tumor-immune microenvironment in triple negative breast cancer revealed by multiplexed ion beam imaging. Cell.

[28] Damond N (2019). A map of human type 1 diabetes progression by imaging mass cytometry. Cell Metab.

[29] Hong SM (2020). Three-dimensional visualization of cleared human pancreas cancer reveals that sustained epithelial-to-mesenchymal transition is not required for venous invasion. Mod Pathol.

[30] Bronsert P (2014). Cancer cell invasion and EMT marker expression: a three-dimensional study of the human cancer-host interface. J Pathol.

[31] Weigert M, Schmidt U, Haase R, Sugawara K, Myers G (2020). Star-convex polyhedra for 3D object detection and segmentation in microscopy.

[32] Santella A (2015). WormGUIDES: an interactive single cell developmental atlas and tool for collaborative multidimensional data exploration. BMC Bioinf.

[33] Wu Y (2013). Spatially isotropic four-dimensional imaging with dual-view plane illumination microscopy. Nat Biotechnol.

[34] Catena R, Özcan A, Jacobs A, Chevrier S, Bodenmiller B (2016). AirLab: a cloud-based platform to manage and share antibody-based single-cell research. Genome Biol.

[35] Schindelin J (2012). Fiji: an open-source platform for biological-image analysis. Nat Methods.

[36] Vincent L, Soille P (1991). Watersheds in digital spaces: an efficient algorithm based on immersion simulations. IEEE Trans Pattern Anal Mach Intell.

[37] Harris CR (2020). Array programming with NumPy. Nature.

[38] Zuiderveld K (1994). Graphics Gems IV.

[39] van der Walt S (2014). scikit-image: image processing in Python. PeerJ.

[40] Napari Contributors (2019). napari: a multi-dimensional image viewer for Python. Zenodo.

[41] Chevrier S (2018). Compensation of signal spillover in suspension and imaging mass cytometry. Cell Syst.

[42] Wolf F, Angerer P, Teis F (2018). SCANPY: large-scale single-cell gene expression data analysis. Genome Biol.

