# Supplementary material for: Three-dimensional imaging mass cytometry for highly multiplexed molecular and cellular mapping of tissues and the tumor microenvironment
Source: Nat Cancer. Author manuscript; Available in PMC 2022 Nov 2. (PMC7613779; doi:10.1038/s43018-021-00301-w)
Supplement: Reporting Summary [file EMS153222-supplement-Reporting_Summary.pdf]

nature research

Corresponding author(s): Bernd Bodenmiller

Last updated by author(s): Sep 6, 2021

Reporting Summary

Nature Research wishes to improve the reproducibility of the work that we publish. This form provides structure for consistency and transparency in reporting. For further information on Nature Research policies, see our Editorial Policies and the Editorial Policy Checklist.

Statistics

For all statistical analyses, confirm that the following items are present in the figure legend, table legend, main text, or Methods section.

n/a	Confirmed	
☒	☐	The exact sample size (*n*) for each experimental group/condition, given as a discrete number and unit of measurement
☒	☐	A statement on whether measurements were taken from distinct samples or whether the same sample was measured repeatedly
☒	☐	The statistical test(s) used AND whether they are one- or two-sided<br>*Only common tests should be described solely by name; describe more complex techniques in the Methods section.*
☒	☐	A description of all covariates tested
☒	☐	A description of any assumptions or corrections, such as tests of normality and adjustment for multiple comparisons
☐	☒	A full description of the statistical parameters including central tendency (e.g. means) or other basic estimates (e.g. regression coefficient) AND variation (e.g. standard deviation) or associated estimates of uncertainty (e.g. confidence intervals)
☒	☐	For null hypothesis testing, the test statistic (e.g. *F*, *t*, *r*) with confidence intervals, effect sizes, degrees of freedom and *P* value noted<br>*Give P values as exact values whenever suitable.*
☒	☐	For Bayesian analysis, information on the choice of priors and Markov chain Monte Carlo settings
☒	☐	For hierarchical and complex designs, identification of the appropriate level for tests and full reporting of outcomes
☒	☐	Estimates of effect sizes (e.g. Cohen's *d*, Pearson's *r*), indicating how they were calculated

Our web collection on statistics for biologists contains articles on many of the points above.

Software and code

Policy information about availability of computer code

Data collection	Commercial Fluidigm software CYTOF Software v.01 available together with the Hyperion imaging system was used for data acquisition.
Data analysis	Raw MCD files from Fluidigm Hyperion imaging system were converted into OME-TIFFs using publicly available IMC data pre-processing pipeline based on imctools v1.0 (https://github.com/BodenmillerGroup/ImcSegmentationPipeline). Image alignment and segmentation including digital pre-processing steps were done in Fiji-ImageJ2-linux64 v1.0. For image registration plugins Register Virtual Stack Slices v3.0.3 and Transform Virtual Stack Slices Release v3.0.0 were used (available from https://github.com/fiji/register_virtual_stack_slices). For single-cell segmentation h-watershed v1.2.1 available from https://github.com/mpicbg-scicomp/Interactive-H-Watershed was used. Prior to segmentation, digital image processing filter CLAHE available in Fiji was applied, followed by another contrast normalization filter also available in Fiji called 'local contrast enhancement' (also provided at https://github.com/axtimwalde/mpicbg/blob/master/mpicbg/src/main/java/mpicbg/ij/integral/NormalizeLocalContrast.java). For post-processing of cell segmentation masks Python scikit-image 1.5.2 package was used. Python3 implementation of Phenograph 1.5.2 was used (available from https://girhub.com/jacoblevine/PhenoGraph) for single-cell clustering. For selecting specific cells of interest directly on images napari v0.4.1rc2 image viewer was used. For 3D rendering of voxel and cell data Agave v1.0.0.1 was used. All custom code that was used for pre-processing images and analyzing single-cell data is available at https://github.com/BodenmillerGroup/3D_IMC_publication

For manuscripts utilizing custom algorithms or software that are central to the research but not yet described in published literature, software must be made available to editors and reviewers. We strongly encourage code deposition in a community repository (e.g. GitHub). See the Nature Research guidelines for submitting code & software for further information.

Data

Policy information about availability of data

All manuscripts must include a data availability statement. This statement should provide the following information, where applicable:

- Accession codes, unique identifiers, or web links for publicly available datasets
- A list of figures that have associated raw data
- A description of any restrictions on data availability

The images and single cell data generated in this study is available at https://doi.org/10.5281/zenodo.4752030

Field-specific reporting

Please select the one below that is the best fit for your research. If you are not sure, read the appropriate sections before making your selection.

☒ Life sciences ☐ Behavioural & social sciences ☐ Ecological, evolutionary & environmental sciences

For a reference copy of the document with all sections, see nature.com/documents/nr-reporting-summary-flat.pdf

Life sciences study design

All studies must disclose on these points even when the disclosure is negative.

Sample size	The sample size was not determined prior to the study as this is a study focusing on a method composed of laboratory and computational workflows. No statistical methods were applied during the data analysis approach and the aim of the study was not biological discovery, thus pre-determining the sample size was not considered necessary. Instead, multiple successful replications of the whole workflow were considered enough to demonstrate that the approach works for different samples.
Data exclusions	No data was excluded from this study. All samples that were stained and imaged were used in this study.
Replication	Replication of this method was succesfully applied to four different samples.
Randomization	Randomization was not applied in this study as this study focuses on a method composed of laboratory and computational workflows. No statistical methods were applied during the data analysis approach and the aim of the study was not biological discovery, thus randomization was not considered necessary. All samples were stained at different times.
Blinding	Blinding was not applied in this study as this study focuses on a method composed of laboratory and computational workflows. The aim of the study was not biological discovery, thus blinding was not considered necessary.

Reporting for specific materials, systems and methods

We require information from authors about some types of materials, experimental systems and methods used in many studies. Here, indicate whether each material, system or method listed is relevant to your study. If you are not sure if a list item applies to your research, read the appropriate section before selecting a response.

Materials & experimental systems

n/a	Involved in the study	
☐	☒	Antibodies
☒	☐	Eukaryotic cell lines
☒	☐	Palaeontology and archaeology
☒	☐	Animals and other organisms
☐	☒	Human research participants
☒	☐	Clinical data
☒	☐	Dual use research of concern

Methods

n/a	Involved in the study	
☒	☐	ChIP-seq
☒	☐	Flow cytometry
☒	☐	MRI-based neuroimaging

Antibodies

Antibodies used	All antibody information is available in Supplementary Table #2, #3 and #4
Validation	Prior to testing antibodies, manufacturer's website and antibody databases were used for choosing antibodies for targets of interest. Antibodies were initially tested using immunofluorescence staining without metal conjugation in lymph node, spleen and breast cancer FFPE sections. Antibodies that revealed expression patterns consistent with the literature were chosen for metal conjugation. After the conjugation, another round of testing was undertaken using IMC with breast cancer FFPE sections, and antibodies that showed staining patterns consistent with the literature and with sufficient signal intensity were utilized. In this step, thanks to multiplexing capabilities of IMC, often already validated antibodies were used in addition to the antibodies being tested to validate

cell type specific staining (i.e., epithelial and different immune cells). Breast cancer FFPE tissues were used for titrating antibodies for final IMC measurements.

Human research participants

Policy information about studies involving human research participants

Population characteristics	Four human breast carcinoma samples were used in this study.
Recruitment	The samples were obtained from the Institute of Pathology and Molecular Pathology at the University Hospital Zurich.
Ethics oversight	Project to use samples was approved by the ethics committee of the Canton Zurich (PB_2016_00811)

Note that full information on the approval of the study protocol must also be provided in the manuscript.
