# Supplementary figures and images for: Three-dimensional imaging mass cytometry for highly multiplexed molecular and cellular mapping of tissues and the tumor microenvironment
Source: Nat Cancer. Author manuscript; Available in PMC 2022 Nov 2. (PMC7613779; doi:10.1038/s43018-021-00301-w)

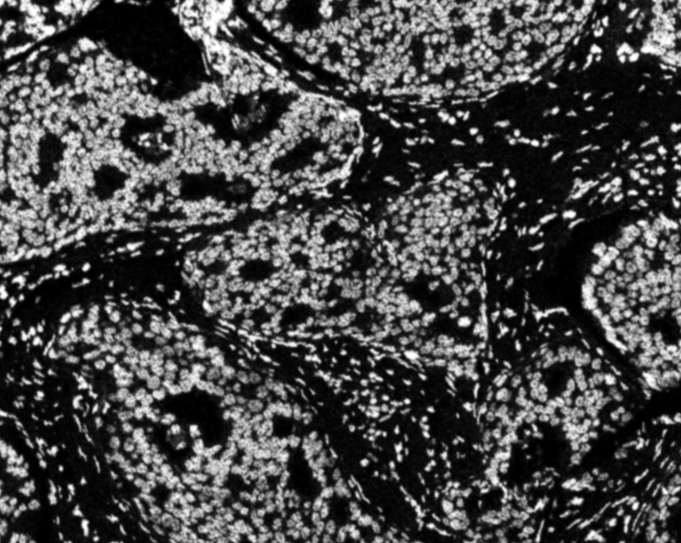

Supplement: Sup_Vdo_1 [file EMS153222-supplement-Sup_Vdo_1.gif]
