# Supplementary material for: Three-dimensional imaging mass cytometry for highly multiplexed molecular and cellular mapping of tissues and the tumor microenvironment
Source: Nat Cancer. Author manuscript; Available in PMC 2022 Nov 2. (PMC7613779; doi:10.1038/s43018-021-00301-w)
Supplement: Supplementary Information [file EMS153222-supplement-Supplementary_Information.pdf]

nature portfolio

https://doi.org/10.1038/s43018-021-00301-w

## Supplementary information

# Three-dimensional imaging mass cytometry for highly multiplexed molecular and cellular mapping of tissues and the tumor microenvironment

In the format provided by the authors and unedited

nature portfolio

https://doi.org/10.1038/s43018-021-00301-w

**Supplementary information**

# Three-dimensional imaging mass cytometry for highly multiplexed molecular and cellular mapping of tissues and the tumor microenvironment

In the format provided by the authors and unedited

Ali HR[1,2], Al Sa'd M[3], Alon S[4], Aparicio S[5,6], Battistoni G[1], Balasubramanian S[1,7], Becker R[8], Bodenmiller B[2], Boyden ES[4], Bressan D[1], Bruna A[9], Burger Marcel[2], Caldas C[9], Callari M[1], Cannell IG[1], Casbolt H[1], Chornay N[3], Cui Y[4], Dariush A[3], Dinh K[10], Emenari A[4], Eyal-Lubling Y[9], Fan J[11], Fatemi A[1], Fisher E[1], González-Solares EA[3], González-Fernández C[3], Goodwin D[4], Greenwood W[1], Grimaldi F[8], Hannon GJ[1], Harris O[8], Harris S[8], Jauset C[1], Joyce JA[12], Karagiannis ED[4], Kovačević T[1], Kuett L[2], Kunes R[10], Küpcü Yoldaş A[3], Lai D[5,6], Laks E[5,6], Lee H[11], Lee M[1,7], Lerda G[1], Li Y[5], McPherson A[5,6,13], Millar N[3], Mulvey CM[1], Nugent F[1], O'Flanagan CH[5], Paez-Ribes M[1], Pearsall I[1], Qosaj F[1], Roth AJ[5,6,14], Rueda OM[9], Ruiz T[5], Sawicka K[1], Sepúlveda LA[11], Shah SP[5,6,13], Shea A[9], Sinha A[4], Smith A[5], Tavaré S[1,10,15], Tietscher S[2], Vázquez-García I[13], Vogl SL[8], Walton NA[3], Wassie AT[4], Watson SS[12], Weselak J[8], Wild SA[1], Williams E[1], Windhager J[2], Xia C[11], Zheng P[11,] Zhuang X[11].

[1] Cancer Research UK Cambridge Institute, Li Ka Shing Centre, University of Cambridge, Cambridge CB2 0RE, UK.

[2] Institute of Molecular Life Sciences, University of Zurich, Zurich 8054, Switzerland.

[3] Institute of Astronomy, University of Cambridge, Madingley Road, Cambridge, CB3 0HA, UK.

[4] McGovern Institute, Departments of Biological Engineering and Brain and Cognitive Sciences, Massachusetts Institute of Technology,Cambridge, Massachusetts, USA.

[5] Department of Molecular Oncology, BC Cancer, part of the Provincial Health Services Authority, Vancouver, BC, Canada.

[6] Department of Pathology and Laboratory Medicine, University of British Columbia, Vancouver, BC, Canada

[7] Department of Chemistry, University of Cambridge, Lensfield Road, Cambridge, CB2 1EW, UK.

[8] Súil Interactive Ltd, Dame Lane, Dublin, UK.

[9] Department of Oncology and Cancer Research UK Cambridge Institute, University of Cambridge, Cambridge, CB2 0RE, UK.

[10] Herbert and Florence Irving Institute for Cancer Dynamics, Columbia University, New York, NY, USA.

[11] Howard Hughes Medical Institute, Department of Physics and of Chemistry and Chemical Biology, Harvard University, Cambridge, MA 02138, USA.

[12] Department of Oncology and Ludwig Institute for Cancer Research, University of Lausanne, Lausanne, Switzerland.

[13] Computational Oncology, Department of Epidemiology and Biostatistics, Memorial Sloan Kettering Cancer Center, New York, USA.

[14] Department of Computer Science, University of British Columbia, Vancouver, BC, Canada.

[15] New York Genome Center, New York, NY, USA.
